# Effect of Organophasphate poisoning among patients reporting at a tertiary healthcare facility of Sindh Pakistan

**DOI:** 10.12669/pjms.343.15000

**Published:** 2018

**Authors:** Shamsuddin Shaikh, Muhammad Saleh Khaskheli, Munazzah Meraj, Hamid Raza

**Affiliations:** 1 Prof. Dr. Shamsuddin Shaikh, MBBS, FCPS (Medicine, FRCPI). Department of Medicine, Peoples University of Medical and Health Sciences for Women (PUMHSW), Nawabshah, Pakistan; 2 Prof. Dr. Muhammad Saleh Khaskheli, MBBS, MCPS, FCPS, MSc Pain Medicine. Department of Anesthesiology, Peoples University of Medical and Health Sciences for Women (PUMHSW), Nawabshah, Pakistan; 3 Dr. Munazzah Meraj, M.Phil, PhD. Assistant Professor, Department of Biochemistry, Peoples University of Medical and Health Sciences for Women (PUMHSW), Nawabshah, Pakistan; 4 Dr. Hamid Raza, MBBS, MCPS, FCPS. Assistant Professor, Head of Department of Anesthesiology, Liaquat University of Medical & Health Sciences, Jamshoro, Pakistan

**Keywords:** Atropine, Organophosphate poisoning, Pralidoxime, Suicidal intention

## Abstract

**Objective:**

To determine the effect of organophosphate poisoning (OPP) among patients at tertiary healthcare, Pakistan.

**Methods:**

This cohort study was conducted over a six-year period (January 2011 to December 2016) of OPP patients admitted to the intensive care unit of Peoples University of Medical & Health Sciences for Women SBA, Hospital Pakistan and their outcome was determined.

**Results:**

Total mortality was 17.39% (84 deaths in 483 patients, excluding those referred to Karachi). Out of these 84 deaths, 65 patients (13.46%) expired due to cardiorespiratory failure, 17 deaths (3.52%) followed due to complication of mechanical ventilation & ICU acquired infection and two deaths (0.41%) occurred due to renal failure. The major cause of poisoning was deliberate self-harm /suicidal intention (93.02%), with gender distribution of female (57.07%) and male (35.95%), followed by unintentional/accidental exposure (6.98%) in males. The intensive supportive treatment, precise and appropriate respiratory care, and adequate amount of atropine and pralidoxime doses are keys to reducing the OPP patient’s mortality.

**Conclusion:**

Organophosphate poisoning (OPP) intoxication is common in female gender. The easy availability of these harmful compounds has resulted in increased mortality either by accidental exposure or most often by the deliberate suicidal attempt.

## INTRODUCTION

Organophosphate compounds (OPCs) are widely used as an agricultural insecticide; this widespread use has resulted in increased mortality and morbidity due to the OP poisoning, specifically in under developed countries.^1^ Globally, approximately three million OPCs poisoning cases have been reported annually, which results in almost 200,000 deaths. According to World Health Organization (WHO), the frequency of mortality due to OPCs poisoning has doubled in developing countries during past 10 years.^1^,^2^ Organophosphate poisoning (OPP) can either be an accidental exposure of OPCs or deliberate suicidal attempt.^2^-^4^ This compound can also lead to the development of cardiac complications which may be serious or fatal, but preventable if diagnosed and brought earliest to hospital and treated well. As limited studies have been done related to the cardiac toxicity therefore frequency, extent and pathogenesis are not well defined, so many physicians may not be fully aware of the complications.^5^,^6^

OPCs may be taken via the oral, respiratory, or transdermal routes. The mechanism of action of OPCs involves the inhibition of acetylcholine esterase (AChE) activity which may cause the accumulation of acetylcholine at neuromuscular junctions and nerve synapses which cause the overstimulation of acetylcholine receptors. This overstimulation of acetylcholine receptors is followed by paralysis of cholinergic synaptic transmission in the central nervous system, in autonomic ganglia at parasympathetic and some sympathetic nerve endings and in somatic nerves.^7^-^9^ The manifestations of OPP may be classified into effects secondary to nicotinic, muscarinic and CNS receptor overstimulation. The overstimulation of muscarinic is manifested as hyperactivity of the parasympathetic system, including bradycardia, meiosis and bronchial glands secretion besides muscle fasciculation, weakness, cramping are nicotinic effects. The effects of central nervous system include respiratory depression, seizures and unconsciousness.^10^-^12^ The mortality rate slightly declined following intensive management as compared to the past where delay in diagnosis and improper treatment caused more deaths.^1^,^11^

The combined therapy of atropine and pralidoxime is the basis of OPP treatment.^1^,^8^,^9^,^13^ Extracorporeal elimination may be a useful adjunctive strategy. However, the use of hemoperfusion in the management of severe OPP remains controversial. According to a recent report, a difference is observed among OPCs insecticides in human self-poisoning, these findings suggest that all OPCs have different toxicity, though the books and publications consider all OPCs as a homogenous entity.^14^,^15^

The rational of the study was that we know that OPP is an important health problem in Pakistan. So, the reduction of the incidence of OPP can be achieved with the control and regulation on sale, appropriate use of such compounds and educating public about the harmful effects of OPP.

## METHODS

This cohort study was conducted over a six-year period (January 2011 to December 2016). The data was collected from intensive care department of PMC hospital patient’s record file. Five hundred seventy-three patients with OPP admitted during the above period were included. The patients were admitted either directly from the emergency department or were shifted later from general medical wards. The diagnosis of OPP solely depended upon: (1) evidence or history of exposure to OPCs within the previous twenty-four hours; (2) the odor of OPCs in the gastric contents; (3) distinctive signs of OPP, including meiosis, fasciculations and excessive salivation (4) improvement of the signs and symptoms of OPP after administration of high doses of atropine and/or pralidoxime. All the above criteria were required to be present in every patient to be included in this study. The resources for the estimation of blood cholinesterase activity were not available

The admission of OPP patients was always through the ED. The patients of OPP in ED were primarily subjected to decontamination procedures, including skin decontamination by removing clothes, washing skin and hair with soap and water, followed by gastric lavage (administration in order to decontaminate the gastrointestinal tract), and cathartics. All the OPP patients were treated with repeated doses of intravenous bolus of 2mg atropine after every 10-15 minutes to achieve tachycardia, xerostomia, mydriasis, flushing and anhydrosis. The OPP patients were then maintained with continuous infusion of atropine 1mg/h according to the features of adequate atropinization such as dry mouth, dry flushed skin, dry tongue, rapid heart rate (120-140/min) and noticeable dilation of pupils. The intravenous pralidoixme at 1-2 g was injected after every six hour for 24-48 hourto all the OPP patients. At intubation or during the period of illness, no patient received any neuromuscular blocking agent except few restless patients on Mechanical Ventilation (MV) who required intravenous midazolam for sedation.

The cause of ingestion, route of poisoning, sex, age and the need for assisted ventilation were recorded at the time of presentation. Data are presented as the mean±SD and the probability values of p<0.05 were considered significant. All statistical analyses were performed using SPSS version 12.0 (SPSS Inc., Chicago, IL, USA).

## RESULTS

Five hundred seventy-three case records (327 females and 246 males) over a period of six years were reviewed who all had severe OPCs poisoning at presentation. The general characteristics of all the OPP subjects included in this study are summarized in Table-I. The mean amount of ingested OP was not estimated. The mean age was 40±5 years for female and 26±4 years for males. The significant difference in the mean age of male and female (p ≤ 0.05) was observed. The major cause of poisoning was deliberate self-harm /suicidal intention (93.02%), female (57.07%) and males (35.95%), followed by unintentional/accidental ingestion (6.98%) in males. The distribution of patients, according to intent of poisoning is shown in Table-I.

**Table-I:** Characteristics of patients with acute organophosphate poisoning.(n=573) Table-I: Characteristics of patients with acute organophosphate poisoning.(n=573)

Characteristics	Value (male)	Value (female)
Age year	40±5	26±4
Duration of hospital stay	2 wks.	03 wks.
Time interval between ingestion and hospital arrival hours	3.9±2.5	4.5±2.6
Death	32	52
Deliberate self-harm (suicidal)	206	327
Unintentional (accidental)	40	--
Ventilation required	150	200
Referred to Karachi	10	80

OPP is a very severe clinical entity and is responsible for high mortality. In the current study, total mortality was about 17.36% (84 deaths in 483 patients) excluding 90 patients referred to Karachi and we don’t know the outcomes of referred patients. Out of these 84 deaths, 65 patients (77.38%) died due to cardiorespiratory failure, 17 deaths (3.52%) followed due to complication of mechanical ventilation & ICU acquired infection and two deaths (0.41%) occurred due to renal failure (Table-II). About ninety percent of the OPP patients presented to doctors within three hours after ingestion, with the mean time interval of about 3.9±2.5 hours. The total amount of atropine administered to various patients significantly varied, according to the requirement. The prescribed mean amount of atropine on the first day was 30.6mg (25-115 mg range), while the mean amount of atropine used in the total treatment of the patients was 136.74 mg (25-650 mg range). The duration of treatment with atropine was 5.5 days (2-20 day range). Pralidoxime, intravenous dose 25-50 mg/kg (1-2 g) given, it is repeated every 6 to 12 hours for minimum one to two days (24 to 48 hours.) until signs resolve. All the patients including in this study received adequate doses of atropine, pralidoxime (biochemical antidote) and combined intravenous atropine and pralidoxime therapy.

**Table-II:** Cause of mortality among patients.(n=483)

Cause of mortality	Male		Female		Total	

	n	%	n	%	n	%
Cardiorespiratory failure	25	5.18	40	8.28	65	13.46
Complication of mechanical ventilation & ICU acquired infection	07	1.45	10	2.07	17	3.52
Renal failure	--	--	02	0.41	02	0.41
Total	32	6.63	52	10.76	84	17.39

Ninety patients referred to Karachi were excluded

In our study, 76.79% (440 patients) have had severe poisoning based on cardiorespiratory failure, renal failure and loss of consciousness. These patients need an assisted ventilation (61.08%) and 15.71% (90 patient) were referred to Karachi. About 69.63% patients were fully recovered and 14.66% (84 cases) died. We observed that the poisonous agent type (carbamate, organophosphate), severity of poisoning, the availability or absence of intensive care facilities and the stage at which treatment was started are the main causative factors for the hospital mortality.

The estimated mean admission time to the ED after the exposure was 3.9±2.5 hours in male, while 4.5±2.6 hours in females. The patient distribution according to year and sex is shown in Table-III. There were 327 (57.07%) female and 246 (42.93%) male patients. The ratio of female/male was 1.33:1. The commonest affected age in female was 26±4 years and in male 40±5 years. Routes of intake were inhalation, transdermal and oral ingestion, no intravenous injection was observed. Oral ingestion (93.02%) observed to be the commonest route of poisoning. A total of 40 (6.98%) patients were exposed to the agent accidentally. But, the most common reason for OPP was the deliberate suicide attempt (93.02%) (Table-I). The average volume of exposed OPCs was not estimated. The most common clinical signs were excessive salivation & bronchial secretions, miosis, respiratory system findings, bradycardia, loss of consciousness, and hypertension. The most frequent sign in OPP was miosis. In our study, bradycardia was seen in 91.27% of the patients.

**Table-III:** OPP affected patient’s distribution according to year, sex and route of poisoning

Year	Sex	Inhalation		Skin		Ingestion		Total	

		n	%	n	%	n	%	n	%
2011	M	04	0.7	01	0.17	25	4.36	30	5.24
	F	--	--	--	--	36	6.28	36	6.28
2012	M	03	0.52	--	--	24	4.19	27	4.72
	F	--	--	--	--	32	5.58	32	5.58
2013	M	10	1.74	02	0.35	37	6.46	49	8.55
	F	--	--	--	--	96	16.75	96	16.75
2014	M	08	1.4	02	0.35	52	9.08	62	10.82
	F	--	--	--	--	76	13.26	76	13.26
2015	M	04	0.7	01	0.17	27	4.71	32	5.58
	F	--	--	--	--	33	5.76	33	5.76
2016	M	04	0.7	01	0.17	41	7.15	46	8.04
	F	--	--	--	--	54	9.42	54	9.42

Total		33	5.76	07	1.21	533	93.02	573	100

On the basis of history, most of these events were attempted intentionally thus suicidal attempt, while few were unintentional thus accidental. Fever of almost 38°C or higher was monitored in the 6.98% of OPP patients within the 24 hours of administration. Muscarinic features (99.65%) were the predominant clinical feature followed by CNS (87.26%) and nicotinic manifestations (78.53%).

Current therapy for OPP includes decontamination, reversal of muscarinic symptoms using atropine, regeneration of acetylcholinesterases using oxime compounds such as pralidoxime (2-PAM), and supportive pulmonary care. In the present study, atropine and pralidoxime treatment was given in the medical ward and ICU along with all supportive care.

## DISCUSSION

OPCs are widely employed as pesticides worldwide. The lack of strict regulations to control the sale of OPCs have made it readily available in the market and resulted in a high rate of accidental and suicidal poisoning.^1^-^4^ The OPP survivals suffer from polyneuropathy which is mostly irreversible. The approximate mortality rate following OPCs ingestion ranges from 15-50%.^1^,^11^,^12^ In the current study, total mortality was about 14.66% (84 deaths in 573 patients). The mortality rate from acute OPP varies between 10-20%, and is generally due to respiratory failure.^11^ The causes of death and mortality rates of the present patients (9.1%) were also in accordance with the literature.^1^,^16^,^17^ We observed that the poisonous agent type (carbamate, organophosphate), severity of poisoning, the availability or absence of intensive care facilities and the stage at which treatment was started are the main causative factors for the hospital mortality. Similar conclusions have also been observed by other investigators.^1^,^5^,^18^,^19^

The most often affected mean age was 26±4 and 40±5 years in females and males respectively. Our findings are in agreement with other studies^11^,^12^.^14^ but some researchers have observed that poisoning with OPCs may occur at all ages.^7^,^8^ Ingestion of OPCs in order to commit suicide is more prevalent in developing countries, and most of the cases reported are suicidal attempts.^2^-^4^ Mild poisoning occurs in case of accidental exposure.^4^,^11^ In some African countries, 40-60% cases of suicidal attempts have been reported.^4^ These high rates of suicidal poisoning and intake by oral means are similar to other studies.^2^,^4^ These high rates may be due to uncontrolled sales and the wide use of these agents all over the country. The signs of OPP may be classified as effects secondary to nicotinic, muscarinic, and CNS receptor overstimulation.^6^ Clinical presentation depends on the specific agent involved, the type of exposure and the quantity absorbed^5^.Miosis was also the most significant sign in the present patient (99.65%). This finding is similar to several studies.^2^,^15^ Other frequent signs were respiratory system findings, tachycardia, emesis, loss of consciousness, hyperhydrosis, hypertension, orhypotension, fasciculation, bradycardia, fever, diarrhea, urinary incontinence, and convulsion. Impaired consciousness was seen in 87.26% of patients. It has been reported this this ratio is 25.27%.^17^ In our study, bradycardia was seen in 91.27% of the patients. This result was contrary to results of other investigators.^16^,^19^ Bradycardia occurs because of muscarinic effects.^13^ Current therapy for OPP includes decontamination, reversal of muscarinic symptoms using atropine, regeneration of acetylcholinesterases using oxime compounds such as pralidoxime (2-PAM), and supportive pulmonary care.^4^,^7^,^20^ Atropine antagonizes the effect of Ach reversing the excessive parasympathetic stimulation by competing for identical binding sites at muscarinic receptors.^14^ Treatment guidelines recommend frequent atropine boluses or infusion therapy until pulmonary secretions are minimized, with endotracheal intubation as needed.^8^,^17^ The most common cause of treatment failure is inadequate atropinization. Inadequate atropinization may contribute to high rates of both aspiration pneumonia and death.^2^,^4^,^16^ Pralidoxime is a biochemical antidote for OPP; its beneficial effects include reactivation of cholinesterase by cleavage of phosphorylated active sites, direct reaction and detoxification of unbounded OPCs, and an endogenous anticholinergic effect.^3^-^5^ This therapy for OPP also applies to the emergency treatment for sarin, a typical drug of chemical weapons of mass destruction.^7^,^14^

## CONCLUSION

The OPP is still an important health problem in Pakistan as in other countries. OPP intoxication is more common in female gender and in these poisonings, suicidal purposes are in the foreground. The key to manage these cases include intensive supportive treatment, precise and appropriate respiratory care, and adequate doses of atropine and/or pralidoxime.

### Author’s contribution

**SS**: Conceptualized and edited the final manuscript.

**MSK**: Literature searche, design the study and approved the final manuscript.

**MM**: Wrote the protocol, first draft of the manuscript and compiled the results.

**HR**: Managed the analyses of the study.
